# Evaluation of usefulness of tongue pressure measurement device for dysphagia associated with treatment of patients with head and neck cancer (ELEVATE)

**DOI:** 10.1097/MD.0000000000033954

**Published:** 2023-06-30

**Authors:** Akihisa Tanaka, Hirokazu Uemura, Takahiro Kimura, Ari Nishimura, Kumiko Aoki, Shintaro Otsuka, Keita Ueda, Tadashi Kitahara

**Affiliations:** a Department of Otolaryngology-Head and Neck Surgery, Nara Medical University, Kashihara, Nara, Japan; b Department of Oral and Maxillofacial Surgery, Nara Medical University, Kashihara, Nara, Japan.

**Keywords:** dysphagia, head and neck cancer, radiotherapy, rehabilitation, tongue pressure measure

## Abstract

Dysphagia is often caused by radiotherapy (RT) in patients with head and neck cancer (HNC), and reduced tongue pressure (TP) is often associated with swallowing dysfunction in the oral stage. However, the evaluation of dysphagia by measuring TP has not yet been established in HNC patients. Herein, we conducted a clinical trial to evaluate the usefulness of TP measurement using a TP-measuring device as an objective indicator of dysphagia induced by RT in HNC patients.

**Methods and Analysis:**

This ELEVATE trial is a prospective, single-center, single-arm, non-blind, non-randomized trial to evaluate the usefulness of a TP measurement device for dysphagia associated with the treatment of HNC. Eligible participants include patients with oropharyngeal or hypopharyngeal cancer (HPC) undergoing RT or chemoradiotherapy (CRT). The TP measurements are conducted before, during, and after RT. The primary endpoint is the change in the maximum TP values from before RT to 3 months after RT. Moreover, as secondary endpoints, the correlation between the maximum TP value and the findings of video-endoscopic and video-fluoroscopic examinations of swallowing will be analyzed at each evaluation point, as well as changes in the maximum TP value from before RT to during RT and at 0, 1, and 6 months after RT.

**Discussion:**

This trial aimed to investigate the usefulness of evaluation by measuring TP for dysphagia associated with HNC treatment. We expect that an easier evaluation for dysphagia will improve rehabilitation programs for dysphagia. Overall, we expect this trial to contribute to the improvement of patients’ quality of life (QOL).

## 1. Introduction

Earlier, surgery was the mainstay of treatment for head and neck cancer (HNC); however, treatment algorithms have changed to include postoperative or definitive chemoradiotherapy (CRT).^[1–3]^ These changes in treatment options have been developed to improve survival, including in recurrent or metastatic settings.

While the number of cancer survivors is increasing, they often suffer from functional disorders, and their quality of life (QOL) declines after treatment.^[4]^ Dysphagia is a significant issue that is relevant to the decline of their QOL, and previous research has demonstrated that it leads to a decline in activities of daily living.^[5]^ As Japan already has a hyper-aging society, preventing dysphagia is a critical issue.

The normal swallowing process is generally classified into oral, pharyngeal, and esophageal stages according to the location of the bolus.^[6]^ In the oral stage, the movement of the tongue, including tongue pressure (TP), plays a key role in transiting the bolus to the oropharynx.^[7–9]^ A lower maximum TP is associated with oral residue and can lead to swallowing dysfunction.^[10]^ Moreover, aging can be a risk factor for lower TP.^[10]^

Radiotherapy (RT) is an important treatment modality for HNC; however, it damages the tumor as well as healthy oral and pharyngo-laryngeal tissues. Dysphagia is developed in approximately half of the patients with HNC as acute and late adverse events (AEs) by RT.^[4,11]^ AEs developed due to RT, such as xerostomia, pain, mucositis, and decreased tongue volume, can induce swallowing dysfunction.^[12,13]^ Regardless, there is no unified view of the correlation between RT and the change in TP, and the incidence and extent of the change have not yet been sufficiently clarified.^[14–16]^ However, RT should decrease TP since it has been clearly shown to induce muscle damage.^[17]^

The usefulness of TP measurement has recently been demonstrated in evaluating swallowing function in patients with neurological diseases and sarcopenia.^[5,18]^ In stroke patients, lower TP, linked to lingual discoordination, can result in swallowing problems in the oral stage.^[19]^ Moreover, TP has been reported to be a good predictor of oral-stage dysphagia.^[20,21]^ The TP measurement device provides objective data that can be easily used to evaluate patients’ swallowing function.^[21,22]^ However, the usefulness of evaluating swallowing function using a TP measurement device has not been established in patients with HNC. Additionally, although videoendoscopic examination of swallowing (VE) and video-fluoroscopic examination of swallowing (VF) are performed as general evaluations for dysphagia, there is a lack of quantitative examinations that evaluate the swallowing function of patients with HNC.^[23,24]^

In this trial, we aim to investigate the usefulness of the TP measurement device as a quantitative evaluation method for dysphagia in HNC patients. Our findings will help improve rehabilitation programs for dysphagia and contribute to the improving the QOL of patients.

## 2. Methods

### 2.1. Study design

This clinical trial, currently ongoing at Nara Medical University Hospital, is a prospective, single-center, single-arm, non-blind, non-randomized clinical trial involving patients with oropharyngeal (OPC) or hypopharyngeal (HPC) cancer who undergo CRT or RT as the standard cancer treatment. Participants will undergo rehabilitation for dysphagia (RD) and several examinations, including TP measurement, VE, VF, cervical computed tomography, ultrasound examination, blood test, chest radiography, electrocardiogram, and respiratory function test. Moreover, AEs will be evaluated according to the Common Terminology Criteria for Adverse Events Version 5.0, as proposed by the National Cancer Institute.

### 2.2. Inclusion criteria

1. Patients with OPC or HPC who underwent CRT or RT as primary treatment, including those who underwent neoadjuvant chemotherapy before CRT or RT.
2. Age range: 18 to 85 years (regardless of gender).
3. Eastern Cooperative Oncology Group performance status of 0 or 1.
4. Patients who have provided written informed consent to participate in this study by themselves or through their surrogates.

### 2.3. Exclusion criteria

1. Patients who cannot understand the investigators’ indications.
2. Patients who cannot maintain the TP probe in an appropriate position using their front tooth.
3. Patients who are unable to apply pressure on the TP probe.
4. Patients with dysphagia caused by past treatment for HNC.
5. Patients with severe dysphagia due to a history of central system disease.
6. Patients who are deemed unsuitable as research subjects by the investigator.

### 2.4. Discontinuation criteria

1. Patients whose TP is difficult to measure due to other conditions (cancer progression and/or AEs) during or after treatment.
2. Patients who decline study participation.
3. Patients who avail the offer to withdraw consent.
4. Patients who do not meet the eligibility criteria after registration.
5. Patients who are unable to come to the hospital due to relocation or other reasons.
6. The entire study is discontinued.
7. The study has been discontinued for other reasons that the investigators deemed appropriate.

### 2.5. Protocol of intervention

CRT or RT is the primary treatment for patients with OPC or HPC. However, concurrent chemotherapy regimens are not regulated. Adjuvant therapies, such as chemotherapy and secondary treatment using immune checkpoint inhibitors, are acceptable if needed.

#### 2.5.1. Method of TP measurement.

After the participants are fully informed, TP is measured 3 times at each evaluation point by a TP measurement device (JMS Tongue pressure measurement device. JMS Co., Ltd.), and the maximum TP values are recorded. After treatment, the change in TP value is analyzed. If TP measurement is impossible, it is recorded as “Impossible” accompanied by a description of the reason.

#### 2.5.2. Data collection.

Patient data will be collected before, during, and after RT, and at 0, 1, 3, and 6 months after the final date of RT (Table 1). TP measurement and VE will be conducted before, during, and after RT and 0, 1, 3, and 6 months after the final date of RT. VF will be conducted before and after RT and at 1, 3, and 6 months after the final date of RT.

**Table 1:**
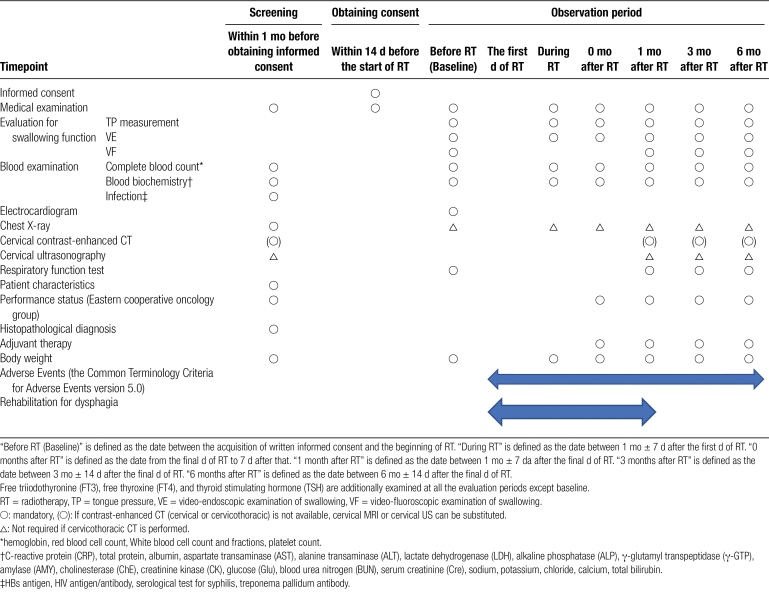
Overview of observation, clinical examination, and evaluation.

“Before RT (Baseline)” is defined as the date between the acquisition of written informed consent and the beginning of RT. “During RT” is defined as the date between 1 month ± 7 days after the first day of RT. “0 months after RT” is defined as the date from the final day of RT to 7 days after that. “1 month after RT” is defined as the date between 1 month ± 7 days after the final day of RT. “3 months after RT” is defined as the date between 3 months ± 14 days after the final day of RT. “6 months after RT” is defined as the date between 6 months ± 14 days after the final day of RT.

#### 2.5.3. Evaluation of VE.

The findings of the VE will be evaluated by the Hyodo score.^[25,26]^

The Hyodo score is defined as follows:

I. The salivary pooling degree at the vallecula and piriform sinuses.
  0: No pooling
  1: Pooling at the only vallecular.
  2: Pooling in the vallecula and piriform sinuses, but no penetration into the larynx.
  3: Pooling in the vallecula and piriform sinuses and penetration into the larynx.
II. The glottal closure reflex induced by touching the epiglottis or arytenoid.
  0: Marked reflex by 1 touch.
  1: Slow and/or weak reflex by 1 touch.
  2: Reflex by 2 or 3 touches.
  3: No reflex despite several touches.
III. The location of the bolus at the swallowing reflex initiation as assessed by “white-out” timing.
  0: Pharyngeal.
  1: Vallecula.
  2: Piriform sinuses.
  3: No swallowing reflex.
IV. The extent of pharyngeal clearance after blue-dyed water is swallowed.
  0: No residue.
  1: Pharyngeal residues remain but are absent after 2 or 3 swallows.
  2: Pharyngeal residues remain but there is no penetration into the larynx.
  3: Pharyngeal residues remain and there is penetration into the larynx.

#### 2.5.4. Evaluation of VF.

The evaluation of the VF has not yet been established. The VF findings will be described from the perspective below.

I. Oral stage.
  1. The contrast medium is kept in oral.
  2. Transition of the contrast medium from oral to pharyngeal.
II. Pharyngeal stage.
  1. Soft palate elevation and velopharyngeal function.
  2. The residual of the contrast medium at the vallecula and piriform sinuses.
  3. Aspiration and expectoration.
  4. Laryngeal elevation.
  5. Laryngeal closure.
  6. Pharyngoesophageal Segment Opening.
  7. Tongue Base Retraction.
III. Esophageal stage.
  1. Esophageal clearance and peristalsis.
  2. Counterflow in the esophagus.
  3. Diseases of and around the esophagus.

#### 2.5.5. Rehabilitation for dysphagia.

The participants will undergo RD to prevent or recover from functional decline in swallowing. RD was routinely scheduled as a protocol intervention during RT. The patients underwent the rehabilitation program in the morning and evening as follows:

1. Ice massage of the oral region (5 sets).
2. Chin push-pull maneuver (10 seconds, 10 sets).
3. Walking (3 minutes).

### 2.6. Endpoints

#### 2.6.1. Primary endpoint.

The primary endpoint was the change in maximum TP value from baseline to 3 months after RT.

#### 2.6.2. Secondary endpoint.

The secondary endpoints are listed as follows:

1. Changes in the maximum TP value from baseline to during RT as well as at 0, 1, and 6 months after RT. We performed the same analysis for the primary endpoint for each evaluation time.
2. The findings of VE by the Hyodo score at each evaluation point.
3. The findings of VF at each evaluation point.

### 2.7. Sample size consideration

The study aims to enroll 40 patients with OPC or HPC who will be treated with CRT or RT over 4 years. The target number of cases was set based on feasibility, given that approximately 12 patients are treated annually with these therapies at the institution. If the average ± SD expected to change in the primary endpoint maximum TP is estimated to be 15.0, the width of the 95% confidence interval (CI) will be appropriately 4.0 ± 2.0 based on a previous study.

### 2.8. Statistical analysis

The “Full Analysis Set” is defined as the population that excludes study participants for whom no data on the maximum TP value are available. Analyses of the primary and secondary endpoints will be performed using the full analysis set.

All analyses will be performed after the data for all endpoints have been collected, reviewed, and fixed.

#### 2.8.1. Primary endpoint.

The average and 95% CI of the changes in the maximum TP value from baseline 3 months after RT will be analyzed as the primary endpoint. Moreover, the ratio of patients with “Impossible” will be calculated.

#### 2.8.2. Secondary endpoint.

1. The average and 95% CI of the changes in the maximum TP value from baseline during RT, as well as 0, 1, and 6 months after RT will be analyzed as secondary endpoints. The ratio of patients with “Impossible” will be calculated as well.
2. The change in the Hyodo score between the baseline and each evaluation point will be analyzed. The averages and 95% CI for the changes will be calculated. Moreover, the scatterplot and Pearson correlation coefficient between the Hyodo score and maximum TP value will be calculated.
3. Dysphagia was evaluated based on the findings of VF at each evaluation point and the ratio of dysphagia among all participants, and the 95% CI of that will be calculated. Moreover, for participants in the group with dysphagia, the average and the 95% CI of both the maximum TP values and Hyodo score will be calculated.

For all the above endpoints, subgroup analyses will be conducted by subject characteristics (e.g., background information) to explore factors that may be useful in determining future research and treatment strategies.

### 2.9. Interim analysis and monitoring

No interim analysis will be scheduled for this trial. However, the trial will be monitored by independent conductors to ensure that it is being conducted safely and in accordance with the protocol and that the data are being collected accurately. An audit will be conducted if the monitoring reveals significant violations of relevant rules and regulations or deviations from the protocol.

## 3. Discussion

The primary objective of this trial was to evaluate the usefulness of the TP measurement device for dysphagia in patients with HNC. The tongue plays a significant role in the oral stage of swallowing, and a decrease in TP may result in dysphagia.^[6–9]^ Therefore, maintaining TP will help prevent dysphagia; thus, better rehabilitation programs for dysphagia have been sought. Currently, a TP measurement device is generally used as a method of measuring TP.^[22]^ In the neurological field, the evaluation of dysphagia by a TP measurement device has already been established, and the correlation between lower TP and dysphagia has been clarified.^[5,18,19]^ However, the utility of assessing dysphagia using TP in patients with HNC has not yet been established. Despite that head and neck tumors often affect swallowing due to being in the oral cavity, pharynx, and larynx, there are currently limited methods for assessing dysphagia associated with HNC treatment.

In clinical practice, VE and VF are usually performed to evaluate dysphagia, and their utility has been established.^[23,24]^ However, these examinations carry a significant risk; therefore, they can cause AEs such as aspiration, pneumonia, and allergy to the contrast medium. Moreover, the VE and VF require practical clinical techniques. Therefore, this trial aimed to investigate the usefulness of evaluation by measuring TP for dysphagia related to HNC treatment.

In this trial, we evaluated the changes in TP affected by RT, since muscle damage by RT has already been reported and decreased TP could result in dysphagia in HNC patients.^[17]^ Thus, this study will include patients with OPC or HPC, in whom the tongue is exposed to radiation during treatment. As TP and swallowing function can be affected by the tumor itself, patients with oral cancer were excluded. Additionally, patients with laryngeal cancer were excluded because the radiation field for laryngeal cancer does not adequately include the tongue. The usefulness of TP measurement as the primary endpoint in HNC patients will be demonstrated. Additionally, the correlation between the change in TP and VE findings, as well as VF, will be analyzed as the secondary endpoint.

Measuring TP as a new method for evaluating swallowing function in HNC patients can provide several benefits. Firstly, the evaluation of swallowing function by measuring TP will enable the reduction of patient burden because the method of TP measurement is noninvasive, simpler, and safer than the VE and VF methods. Also, it allows easy assessment of swallowing function anywhere without the need for endoscopy or an X-ray device. Secondly, the TP measurement device provides objective and quantitative data that can be easily shared with medical staff to determine the extent of a patient dysphagia. Thirdly, objective swallowing data also helps in evaluating the effectiveness of RD. We expect that an easier evaluation of RD effectiveness will improve rehabilitation programs for dysphagia.

Overall, we expect this trial to contribute to the improvement of patients’ QOL.

## Acknowledgments

We thank the staff of the Department of Otolaryngology-Head and Neck Surgery of Nara Medical University and the Institute for Clinical and Translational Science of Nara Medical University Hospital, especially Masahiro Isogawa, for assisting in refining the protocol.

## Author contributions

**Conceptualization:** Akihisa Tanaka, Hirokazu Uemura, Kumiko Aoki.

**Data curation:** Akihisa Tanaka, Hirokazu Uemura, Takahiro Kimura, Ari Nishimura.

**Formal analysis:** Keita Ueda.

**Funding acquisition:** Akihisa Tanaka, Hirokazu Uemura.

**Investigation:** Akihisa Tanaka, Takahiro Kimura, Ari Nishimura, Kumiko Aoki.

**Methodology:** Hirokazu Uemura, Kumiko Aoki.

**Project administration:** Hirokazu Uemura.

**Supervision:** Shintaro Otsuka, Tadashi Kitahara.

**Writing – original draft:** Akihisa Tanaka.

**Writing – review & editing:** Hirokazu Uemura, Takahiro Kimura, Ari Nishimura, Kumiko Aoki, Shintaro Otsuka, Keita Ueda, Tadashi Kitahara.
